# SIMAT: GC-SIM-MS data analysis tool

**DOI:** 10.1186/s12859-015-0681-2

**Published:** 2015-08-19

**Authors:** Mohammad R. Nezami Ranjbar, Cristina Di Poto, Yue Wang, Habtom W. Ressom

**Affiliations:** 1 Department of Electrical and Computer Engineering, Virginia Tech, Arlington, VA USA; 2 Department of Oncology, Georgetown University, Washington DC, USA

**Keywords:** Selected ion monitoring, Gas chromatography-mass spectrometry, Data analysis

## Abstract

**Background:**

Gas chromatography coupled with mass spectrometry (GC-MS) is one of the technologies widely used for qualitative and quantitative analysis of small molecules. In particular, GC coupled to single quadrupole MS can be utilized for targeted analysis by selected ion monitoring (SIM). However, to our knowledge, there are no software tools specifically designed for analysis of GC-SIM-MS data. In this paper, we introduce a new R/Bioconductor package called SIMAT for quantitative analysis of the levels of targeted analytes. SIMAT provides guidance in choosing fragments for a list of targets. This is accomplished through an optimization algorithm that has the capability to select the most appropriate fragments from overlapping chromatographic peaks based on a pre-specified library of background analytes. The tool also allows visualization of the total ion chromatograms (TIC) of runs and extracted ion chromatograms (EIC) of analytes of interest. Moreover, retention index (RI) calibration can be performed and raw GC-SIM-MS data can be imported in netCDF or NIST mass spectral library (MSL) formats.

**Results:**

We evaluated the performance of SIMAT using two GC-SIM-MS datasets obtained by targeted analysis of: (1) plasma samples from 86 patients in a targeted metabolomic experiment; and (2) mixtures of internal standards spiked in plasma samples at varying concentrations in a method development study. Our results demonstrate that SIMAT offers alternative solutions to AMDIS and MetaboliteDetector to achieve accurate detection of targets and estimation of their relative intensities by analysis of GC-SIM-MS data.

**Conclusions:**

We introduce a new R package called SIMAT that allows the selection of the optimal set of fragments and retention time windows for target analytes in GC-SIM-MS based analysis. Also, various functions and algorithms are implemented in the tool to: (1) read and import raw data and spectral libraries; (2) perform GC-SIM-MS data preprocessing; and (3) plot and visualize EICs and TICs.

**Electronic supplementary material:**

The online version of this article (doi:10.1186/s12859-015-0681-2) contains supplementary material, which is available to authorized users.

## Background

Gas chromatography coupled with mass spectrometry (GC-MS) is one of the technologies widely used for qualitative and quantitative analysis of small molecules. The technology is useful in studies that aim to evaluate the metabolite levels in biofluids and tissues. The use of electron impact (EI) ionization enables the instrument to generate mass spectra for different compounds by shattering biomolecules in multiple fragments.

Although GC-MS instruments are mostly used for untargeted analysis of metabolites, selected ion monitoring (SIM) allows users to monitor a subset of fragments with their related mass values in a certain retention time (RT) range for a set of targets. This targeted method is expected to lead to increased sensitivity compared to full-scan, as the mass spectrometer monitors a small fraction of the mass range in the former case. The fragments for a targeted method are usually selected in such a way that they are the most unique choices per target. The most unique fragment is used for quantification, whereas the remaining fragments are used as qualifiers to help with the identification. However, it is not always easy to pick appropriate fragments, because of the presence of background analytes overlapping with the targets of interest.

Several tools have been developed to process the raw GC-MS data. These include freely available tools such as automated mass spectral deconvolution and identification system (AMDIS) [1] from NIST, MetaboliteDetector [2], TagFinder [3], and TargetSearch [4] or commercial software such as Mass Profiler Professional (MPP) [5] by Agilent and ChromaTOF by Leco [6].

Although many of these tools can be used for analysis of GC-SIM-MS data, some additional features are desired. For example, after peak deconvolution, it is considerably effective to define only a subset of the fragments from the same chromatographic peak to be used for spectral matching. Although MetaboliteDetector allows manual definition of such mass ranges, one cannot define the mass for each compound separately or at least over a certain range of RT. Also, the values can not be imported from a table at once. Moreover, the user is required to re-enter the values when repeating the same analysis.

The limitation to define target-specific fragments leads to interfering chromatographic peaks from fragments that do not belong to the analyte of interest. The goal is to ensure that appropriate fragments are selected, particularly the unique mass to be used for the purpose of quantification. Due to a lack of software tools, the selection of such fragments is typically performed manually by the user. Tools such as TagFinder [3] and TargetSearch [4] limit the peak detection, peak grouping, and analyte identification to apex of the peaks. Although, there are some advantages when working with apex of the peaks in case of interference, in some cases, based on the overlapping pattern of the interfering compound, the location and the intensity of the apex of particular fragments may change significantly.

Even if other tools such as XCMS [7] can be used to perform peak detection and deconvolution, they lack algorithms required for peak grouping followed by spectral matching which are necessary for identification and alignment steps in GC-MS data analysis.

In addition as in many SIM analyses only a few fragments are selected to be monitored, the regular weighted dot-product used for calculation of spectral matching is not adequate to pinpoint most of the fragment peaks. Also, for a large sample size, the manual curation of error occurred in the peak detection and analyte identification steps is not feasible [4]. Therefore, a tool capable of automatically detecting and estimating the intensities of fragment peaks with overlapping components is desired.

In a response to this demand, we introduce a new tool for analysis of GC-SIM-MS data which addresses the aforementioned shortcomings of available software. Our tool provides guidance to users on the selection of the appropriate fragments before running the experiment based on a pre-specified library of background analytes. Also, the user can provide his or her in-house library including potential background analytes or any combination of libraries at hand. Moreover, we use a combination of both apex and EIC information when performing peak detection to improve the estimation of the similarity scores.

Although GC-MS instruments are quite reproducible in terms of RT, it is very helpful to use retention index (RI) as a standard method for the purpose of alignment and identification. A mixture of RI standards will be used for this purpose where the RT differences between the standards are known. While several RI standard mixtures, such as FAME and Alkanes are available, users can create their own set of RI standards to use them with their in-house libraries.

## Implementation

SIMAT has been implemented as an R/Bioconductor package. The run time is shorter than analysis of typical GC-MS data acquired in full-scan mode, because the tool does not search for chromatographic peaks over the entire span of RT.

### Inputs

First, tools such as HP ChemStation by Agilent and ChromaTOF by Leco are used to convert the data to the standard netCDF format. As GC-SIM-MS analysis is a targeted method, the RT and spectral information of the targets should be provided by the user. In addition, a library can help improve the selection of the appropriate fragments. Also user may provide the preferred fragments per analyte to be used as quantifier mass. However, if user does not provide this information, SIMAT will decide based on other criteria outlined in following sections.

### Algorithm

**Fig. 1:**
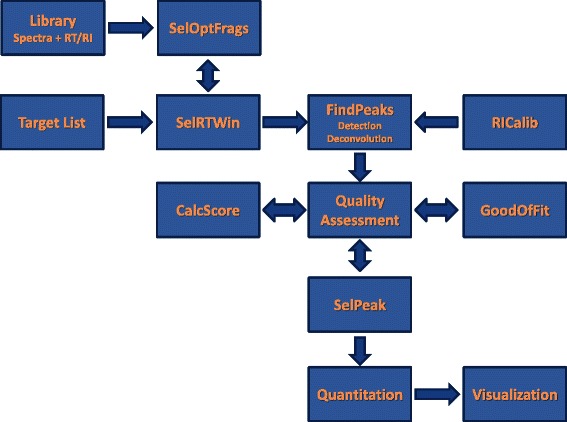
SIMAT pipeline. The SIMAT pipeline flowchart for experimental design and analysis of GC-SIM-MS data

As shown in Fig. 1, SIMAT has several functionalities including optimal fragment selection, baseline correction, peak detection and deconvolution, RI calibration (optional, requires RI standards information), metabolite identification, quantification, and visualization.

#### Optimal fragment selection

A list of quantifier and qualifier fragments is required per target analyte prior to analysis by GC-SIM-MS. The most unique fragment from the spectrum of each target is usually selected manually. This is a time consuming task where users are prone to make errors particularly when overlapping fragments are involved. We define two analytes as overlapping if their RTs or RIs are within a pre-specified tolerance criterion. The overlapping analytes can be determined from the target list, by using a comprehensive mass spectral library such as Fiehn [8] and NIST [9], or an in-house library including known and unknown background analytes from the experimental runs.

Although the uniqueness of the fragments is the most reasonable feature to decide on the appropriate quantifiers and qualifiers, the relative intensity of the fragments should be taken into account as an important criterion too. In other words, a fragment *F* may be unique for analyte *A* considering the overlapping analytes *B* and *C*, but its relative intensity in the spectrum of the analyte *A* may be too small, e.g. less than 5 % of the top three most abundant fragments. Therefore, *F* is not a good choice as a quantifier of *A*.

Another important consideration is the grouping of targets in RT windows. This is because the intensity could decrease if too many targets are inserted in one window. Therefore, there is a limit on the number of targets per RT window based on the mass and total number of fragments for each target in that window.

By providing a library including RTs or RIs for each compound, the tool can come up with a sorted list of preferred fragments and RT windows (segments) for a list of targets. However we recommend the user to run several technical and analytical replicates of exemplar samples, to review the background signals and select candidate fragments for each analyte of interest. This initial evaluation can be done manually or automatically using the optimal fragment selection function in SIMAT.

By comparing the mass of each fragment for each target with those of other targets or library compounds within a specified range of RT or RI (when provided), we calculate the penalty score as below to sort the fragments for each target:

(1) $$\begin{array}{@{}rcl@{}} P_{i,j} = \frac{1}{a_{i,j}} \sum\limits_{k=1}^{\mathcal{O}_{m_{i,j}}} \exp \left(-\frac{|r_{i} - r_{k}|}{\delta R} \right) \end{array} $$

where *m*_*i*,*j*_ and *a*_*i*,*j*_ are the mass and intensity of the *j*th fragment from the spectrum of the *i*th target, *r*_*i*_ is the RI of the *i*th target, and *k* is the index of all fragments sharing the same mass of that fragment, i.e. $\mathcal {O}_{m_{i,j}}$. Therefore, a fragment with a lower *P* is preferred. Here, *δ* *R* is the parameter to control the tolerable range of the difference in RI. Thus, the larger *δ* *R* means less sensitivity in RI mismatch.

**Fig. 2:**
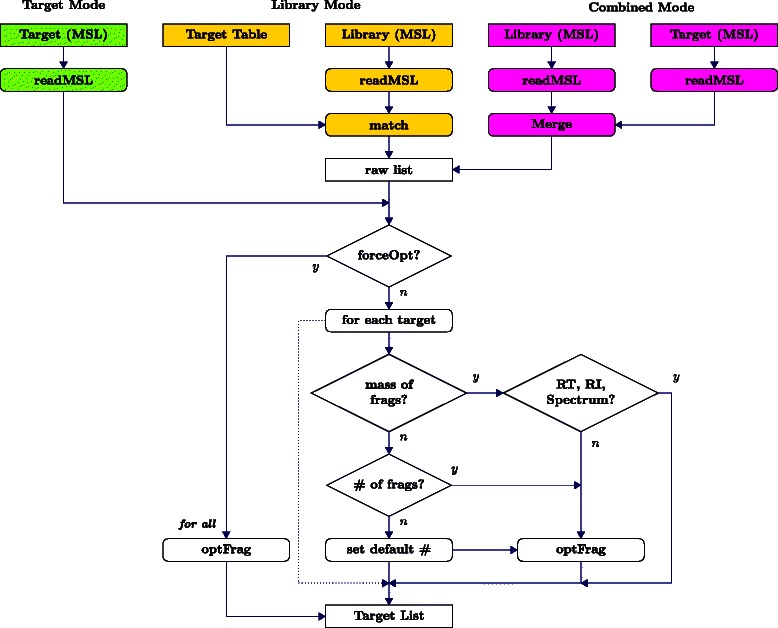
Optimization flowchart. Flowchart of target list processing and fragment selection by optimization in three different modes
- Library mode: The user provides a target table, e.g. in csv format, and a library in MSL format. The user may provide either the mass of fragments or the number of desired fragments. Alternatively, the user can provide just the name of the target. In this situation, the tool retrieves the intensity, RT and RI for which the fragment mass are provided by the user. Optimization is done for those targets with no mass information to select the top fragments based on the desired number. If the desired number is not provided, the algorithm uses an adjustable default value.
- Combined mode: The user can provide both targets and the library in MSL format. In this case, first, an aggregated library is made by merging the target file and the provided library. Then, the algorithm follows as the previous case with the list of targets obtained from the MSL file.

SIMAT provides users with flexible options regarding the input method of target information and selection of fragments through optimization. As shown in Fig. 2, the user can choose among three modes, i.e. target, library, and combined:

- Target mode: The user provides the targets as an MSL file including RT, mass, and intensity for each fragment.

In the above three modes, by setting a flag in the related function, the user may force the optimization algorithm to be applied to all entries whether the mass of fragments are provided or not. However, in this case the algorithm still follows the desired number of fragments if provided by the user, otherwise, a default value is used.

The optimization module is the most time consuming step in SIMAT. The running time depends on the number of targets as well as the number of compounds in the library and the complexity of their spectra. For a target list of size 50 and a library with 1000 compounds, the optimization may take several minutes on a desktop computer.

#### Baseline correction

Baseline correction is performed at the beginning of the SIMAT processing pipeline. This is done for each fragment separately, as each monitored mass may have a different level of background signal. Alternatively, baseline correction can be performed using the tools applied for data conversion.

#### Peak detection and deconvolution

Before peak detection, a smoothing filter is used. Then, the profiles of the quantifiers are utilized to locate and evaluate the reliability of the corresponding fragment peaks for each target. This is done by comparing the similarity scores of all candidate peaks with the target analyte using spectral matching and RI distance. Therefore, all candidate peaks in vicinity of the expected RT of the target, are evaluated to pinpoint the best fit.

After peak deconvolution, a peak shape quality score (QS) is assigned to each retrieved EIC by measuring its goodness of fit using a Gaussian shape. The generated QS is used to evaluate the reliability of identification and quantification results to help us decide whether or not to include the EIC of a fragment in calculating the similarity score or estimating the intensity of the analyte.

Because of the modular design of the tool, the peak detection algorithm can be replaced with any function the user prefers as long as the superseded version provides the location of the fragment peaks in a numeric vector.

#### Retention index calibration

We recommend using RI standards to allow RI calibration as it helps improve peak detection and analyte identification, leading to less false positives. The calibration is done by regressing a piecewise linear model using the RT values of the RI standards. Then, the learned linear model is used to estimate the RI of the target analytes. This information will be used in our peak detection step, i.e., by comparing the RI of detected analyte to those of targets in the library, analyte identification can be performed more reliably. Also, because of the modular design of the tool, the RI calibration function can be any function with the same properties provided by user.

Although SIMAT provides an RI calibration solution, the user is not limited to using this option. If the user does not provide any RI information, the RTs are directly used to perform the analysis. In this case, it is recommended that the user provides the length of the search window around the expected RT of each target. An estimated value can be obtained by running few samples in full scan (profiling mode) and averaging the shifts in RT of some known compounds. If this value is not provided by user, SIMAT uses a default value of 4 sec.

#### Analyte identification

The regular weighted dot-product-based similarity scores are not adequate for analyte identification based on SIM data in which very few fragments are present for each analyte. Therefore, we used a mixed score, i.e. Eq. 2, based on two spectral matching scores: (i) the weighted dot-product of spectra as shown in Eq. 3; (ii) the average pairwise intensity comparisons between two spectra using the ratios of all fragments as stated by Eq. 4:

(2) $$\begin{array}{@{}rcl@{}} S_{1,2} = \frac{N_{1} S_{DP} + N_{1\cap 2} S_{PR}}{N_{1} + N_{1\cap 2}}  \end{array} $$

(3) $$\begin{array}{@{}rcl@{}} S_{DP} = \frac{\left(\mathbf{m}_{1}^{T}\mathbf{m}_{2}\right)^{w_{m}}\left(\mathbf{a}_{1}^{T}\mathbf{a}_{2}\right)^{w_{a}}}{\sqrt{\left(\sum\limits_{i=1}^{M} m_{1,i}^{2w_{m}}a_{1,i}^{2w_{a}}\right)\left(\sum\limits_{i=1}^{M} m_{2,i}^{2w_{m}}a_{2,i}^{2w_{a}}\right)}}  \end{array} $$

(4) $$\begin{array}{@{}rcl@{}} S_{PR} = \frac{1}{N_{1\cap2}} \sum\limits_{k=1}^{N_{1\cap2}} \min \left(\alpha_{k}, \frac{1}{\alpha_{k}}\right)  \end{array} $$

where *N*_1_ and *N*_1∩2_ are respectively the number of fragments in the unknown analyte’s spectrum and the number of shared fragments between the unknown analyte and the reference analyte from the library. Also, **m** and **a** are vectors of fragments’ mass and intensities, respectively. The *w*_*m*_ and *w*_*a*_ are the weights associated with mass and intensity and *M* is the maximum possible number of fragments. Moreover:

(5) $$\begin{array}{@{}rcl@{}} \alpha_{k} = \frac{a_{2,k}}{a_{2,k-1}}\frac{a_{1,k}}{a_{1,k-1}} \end{array} $$

which means depending on the size of the product of the ratios of paired intensities, we always select the value to make sure all summation terms in Eq. 4 are non-negative values less than 1.

The original idea for the mixed score was introduced in [10]. However, we improved the performance of the second score by using all pairwise ratios when performing the comparison between two spectra:

(6) $$\begin{array}{@{}rcl@{}} S_{PR}^{\ast} = \frac{1}{N_{1\cap2}^{\ast}} \sum\limits_{\substack{k=1\\ \ell>k}}^{N_{1\cap2}} \min \left(\alpha_{k,\ell}^{\ast}, \frac{1}{\alpha_{k,\ell}^{\ast}} \right) \end{array} $$

where:

(7) $$\begin{array}{@{}rcl@{}} N_{1\cap2}^{\ast} = \left(\substack{N_{1\cap2}\\ 2} \right) \end{array} $$

and:

(8) $$\begin{array}{@{}rcl@{}} \alpha_{k,\ell}^{\ast} = \frac{a_{2,k}}{a_{2,\ell}}\frac{a_{1,k}}{a_{1,\ell}} \end{array} $$

Different overlapping patterns may occur depending on the distance of interfering compounds and their relative intensities leading to different and some times quite incomparable spectral matching scores. Therefore the user can choose to use either the AUC-based or apex-based scores. Finally, it is possible to use a weighted combination of the two where the default weights are 0.5, but they can be modified by the user:

(9) $$\begin{array}{@{}rcl@{}}  S_{1,2}^{C} = w S_{1,2}^{Apex} + (1-w) S_{1,2}^{AUC} \end{array} $$

where 0≤*w*≤1.

The following equation is used to calculate the RI similarity score [2]:

(10) $$\begin{array}{@{}rcl@{}}  S_{1,2}^{R} = \exp\left(-\frac{|r_{1} - r_{2}|}{\delta R}\right) \end{array} $$

where *δ* *R* is the decay rate of the difference between RIs. The overall similarity score is then calculated based on the product of $S^{C}_{1,2}$ from Eq. 9 and $S^{R}_{1,2}$ from Eq. 10. Because of the modular design of the software, the penalty function can be replaced by any other function in case required.

#### Quantification

When the analyte of interest is detected reliably in previous steps, the related intensity is calculated based on both the apex of fragments and the AUC of each fragment using the corresponding EIC. Therefore, the tool provides the intensities for all quantifier and qualifier fragments per analyte. However, it is recommended to use the intensities from the quantifier mass for relative quantitative comparisons.

#### Visualization

The pipeline provides functions which enables the user to plot the TIC of a run, or a set of runs. The tool is also able to visualize the profile of any target in an individual run by plotting the EIC of all corresponding fragments. The EIC plots also include a subplot showing a pseudo chromatographic peak based on the expected EICs for all fragments and the spectral information of the targets. The mass of each fragment is shown while plotted in different colors. In addition, two vertical dashed lines show the expected and attained RTs of the target respectively.

### Outputs

The primary output of the tool is a table including the name of the target analytes with their quantitated intensities across runs. In addition, if the user chooses to use optimal fragment selection, a list of analytes with their preferred fragments will be provided to be used in the GC-SIM-MS data acquisition.

The tool also provides the EICs of the detected analytes where the user can visually evaluate them. This can be done for individual analytes in a specific run, or by providing a range of runs (using run names or run orders) and a range of compounds (or compound names) from the list of targets. The generated EICs can be used for further analysis, such as visual inspection of randomly selected targets to ensure the accuracy of the data preprocessing step.

## Results and discussion

To evaluate the performance of SIMAT, we used two experimental data sets, Data Set 1 and Data Set 2. Data Set 1 is from a targeted metabolomic experiment that includes 86 subjects representing two disease groups. Data Set 2 contains 40 runs from a spike-in experiment using mixtures of four internal standards. We analyzed these two data sets with AMDIS+MPP, MetaboliteDetector, and SIMAT. The evaluation performance was based on the number of correctly detected analytes. The parameter settings are provided in Additional file 1. Also, we illustrate different functions in SIMAT using a demo data set (DSdemo). All three data sets were analyzed by GC-SIM-MS using the metabolite extraction and data acquisition methods described in the following sections.

***Metabolite extraction*** Plasma metabolites were extracted by adding 1mL of working solution composed of acetonitrile, isopropanol, and water (3:3:2) containing myristic acid d27 to 30 *μ*L of plasma. After vortexing, samples were centrifuged at 14,500 g for 15min at room temperature. The supernatant was then divided into two, 460 *μ*L each, for analysis by each of the GC-MS system.

We derivatized the dried samples in each batch prior to injection following a two-stage process of oximation followed by trimethylsilylation (TMS) [11, 12]. Briefly, 20 *μ*L of a 20 mg/mL methoxyamine hydrochloride in pyridine was added to the dried extracts, vortexed and incubated at 80 °C for 20min. After returning the samples at room temperature, 91 *μ*L of MSTFA + RI standards was added, vortexed and incubated at 80 °C for 20 minutes. Samples were then centrifuged at 14,500 rpm for 15min, and 60 *μ*L of the supernatant was transferred into 250 *μ*L clear glass autosampler vials. The labeled internal standards were purchased from CDN isotopes and RI standards were purchased from TCI chemicals.

***Data acquisition*** The data sets were acquired by analyzing the metabolite extracts using a 7890A Agilent Gas Chromatograph coupled with a 5975C single quadrupole Mass Spectrometer (GC-qMS). Agilent J&W DB-5MS column (30m × 0.25mm × 0.25 *μ*m film 95 % dimethyl 5 % diphenyl polysiloxane) + 10m Duraguard Capillary column was used. Prior to GC-MS analysis, retention time locking (RTL) was performed using myristic acid d27 at 16.727min. The samples were then injected in splitless mode, with the injection port held at 250 °C. The initial oven temperature was held at 60 °C for one minute and then ramped at 10 °C/min to 325 °C and held for 10min. The post run was one minute to allow the oven cool down to 60 °C. MSD transfer line was held at 290 °C, ion source at 250 °C and the mass analyzer at 150 °C. The GC-qMS data were acquired in 37.5min with 5.9min solvent delay at normal scan rate in the mass range 50-600Da.

### Data Set 1: targeted metabolomic experiment

Data Set 1 was obtained by a targeted analysis of 69 metabolites in plasma from 86 subjects using an Agilent GC coupled with a single quadruple MSD mass spectrometer in SIM mode.

**Fig. 3:**
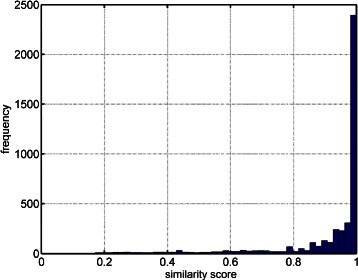
Similarity score histogram. Histogram of similarity scores for targets detected in Data Set 1. As shown most of the targets had similarity score above 0.8 threshold

**Fig. 4:**
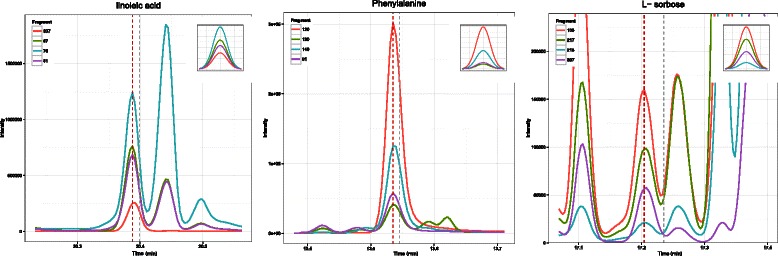
Target EIC. Extracted ion chromatogram of monitored fragments for a target in Data Set 1. Using a modified spectral matching score in combination of RI calibration, SIMAT is able to pick the correct chromatographic peak among multiple cases

Table 1 shows a comparison between different tools based on the number of peaks detected with a similarity score higher than a threshold. As shown in the table, SIMAT leads to detection of more metabolites compared to other tools for the same threshold of similarity score. For example, SIMAT detected 85.3 % of the targets with a score higher than 0.8 out of 1.0 while using AMDIS+MPP and MetaboliteDetector, only 34.6 % and 53.7 % of the analytes were detected for the same threshold of 0.8, respectively. At best, we were able to detect 61.9 % of metabolites with a score higher than 0.6 in all runs using AMDIS+MPP. Similarly, using MetaboliteDetector, we detected 70.2 % of total analytes in all runs with a score higher than 0.6. Figure 3 illustrates the histogram of the similarity scores of the targets detected by SIMAT. Also, Fig. 4 shows an example of the EIC obtained by using SIMAT.

**Table 1:** Percentage of detected targets for different similarity score thresholds for Data Set 1

Score threshold	0.60	0.70	0.80
SIMAT	92.3	89.0	85.3
AMDIS + MPP	61.9	47.4	34.6
MetaboliteDetector	70.2	65.5	53.7

**Fig. 5:**
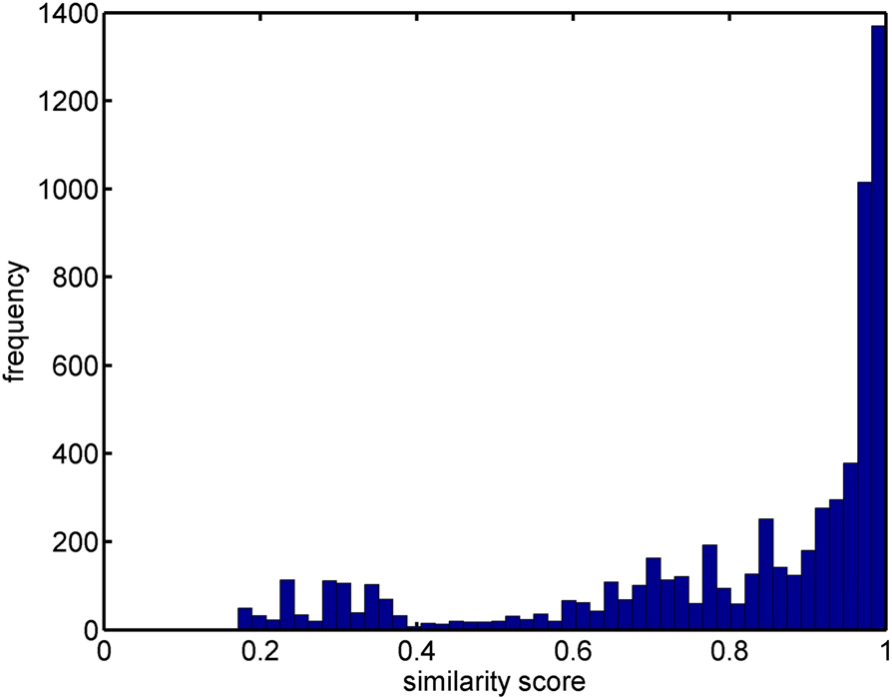
Similarity score histogram. Histogram of similarity scores for detected targets when default fragments from the Fiehn SIM library were used with Data Set 1. Compared to Fig. 3, the scores are shifted to the left, indicating that the identification is not as confident as the former case, where fragments are selected by optimization

**Fig. 6:**
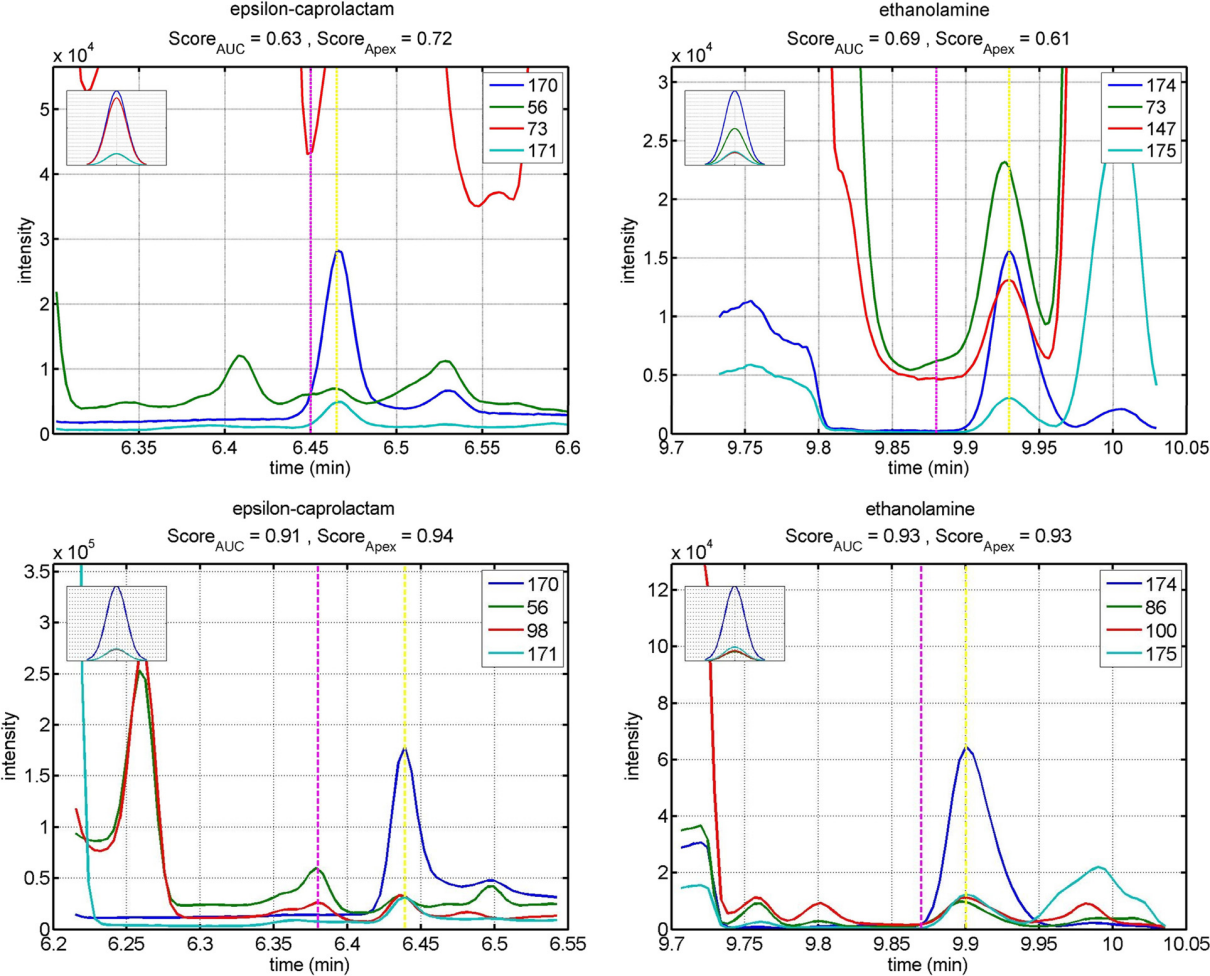
EIC comparison. Comparison between the EICs of two analytes before (top row) and after (bottom row) using optimal fragment selection in Data Set 1. This shows the effectiveness of choosing appropriate fragments by using optimization techniques while a pre-specified library of background compounds is provided by the user

Figure 5 illustrates the histogram of the targets when optimal target selection is not used. Figure 6 shows two analytes to demonstrate the power of using optimal fragment selection versus choosing fragments based on the Fiehn SIM library. As illustrated, the selection of the fragments based on default values suggested by the library is not always efficient. In fact, in some cases, it may even make the detection of the analytes very difficult which subsequently may lead to inaccurate quantification of the targets.

### Data Set 2: spike-in experiment

Data Set 2 was acquired by the same instrument as Data Set 1. It consists of 40 runs including 39 runs of three technical replicates of four standards (Table 2) at 13 different concentration levels ranging from 1× to 2^−12^×, where the base concentration 1× was specifically determined based on each standard. The standards were spiked-in plasma samples. This allows us to evaluate the power of our tool in quantification of the targets in the presence of background analytes. Also, for quality assessment, a sample including all four standards with the base concentration was ran at the end of queue in full scan.

**Table 2:** List of spike-in internal standards in Data Set 2

Internal standard	Quantifier	Qualifier 1	Qualifier 2
glutamic acid d5	161	235	263
tryrosine d2	218	282	181
myristic acid d27	312	76	135
phenylalanin-phenyl d5	219	200	274

**Fig. 7:**
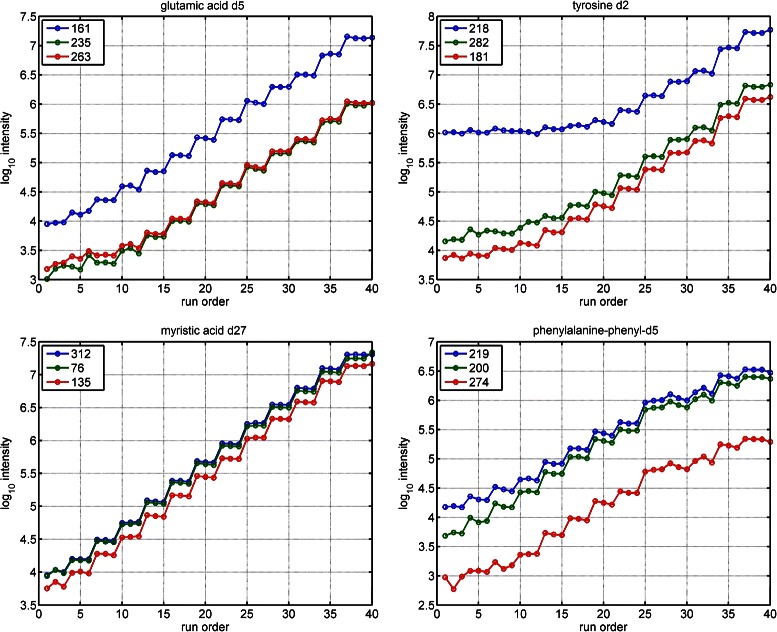
Spike-in experiment. Reproducibility of the spike-in standards over a wide range of concentration levels in Data Set 2

Figure 7 shows the log intensity of the quantifier and qualifier fragments for the four standards determined by SIMAT. As shown in the figure, because of existing background, some fragments hit the baseline concentration and remain almost flat below a certain level. For instance, in the case of tyrosine d2, the fragment with mass 218 is not a good choice, as its trend shows that another analyte sharing the same fragment exists in vicinity of tyrosine’s elution time. While the background may originate from known or unknown compounds, this example shows that appropriate choice of fragments for monitoring is very important, mainly for low abundant analytes.

In summary, using SIMAT, we were able to detect all the standards in all runs at each concentration level considered in this study using 0.8 as the cutoff score. In comparison, on the basis of the number of missing values and the confidence of the similarity scores, AMDIS+MPP and MetaboliteDetector detected 52 % and 64 % of the total target analytes across all runs with a similarity score threshold of 0.8, respectively.

### Data processing example

Here, we demonstrate how SIMAT can be used for processing of GC-SIM-MS data through a demo data set embedded in the package. The list of analytes in this data set is shown in Table 3. After installation, we start by loading the package and example data sets included in the SIMAT library:

**Table 3:** List of 17 analytes in the package’s embeded data set

Analyte	QF	QL _1_	QL _2_	QL _3_	RI
epsilon-caprolactam	56	98	170	171	703.8
norvaline	72	73	74	75	791.7
valine	144	73	147	218	897.5
urea	189	66	74	190	920.2
phosphoric acid	300	211	73	314	954.8
proline	59	142	143	216	978.5
threonine	117	218	219	291	1061.8
trans–hydroxy-L-proline	230	231	304	147	1134.2
aspartic acid	188	202	218	232	1191.3
phenylalanine	91	120	130	146	1223.4
glutamic acid d5	132	147	251	252	1289
glutamic acid	128	156	246	247	1292.1
lauric acid	257	129	132	57	1326.6
arabitol	217	205	117	319	1389.4
sorbose	103	217	218	307	1549.7
linoleic acid	67	75	81	337	1880.7
arachidic acid	117	129	132	145	2103.9

QF: quantifier, QL: qualifier, RI: retention index (FAME)

**Fig. 8:**
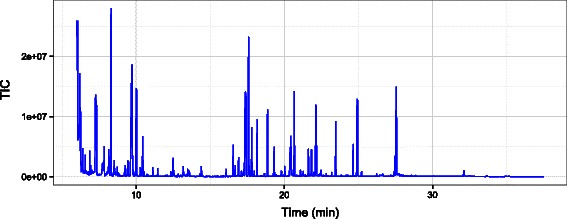
TIC example. An example of total ion chromatogram (TIC) of a run visualized by SIMAT

**Fig. 9:**
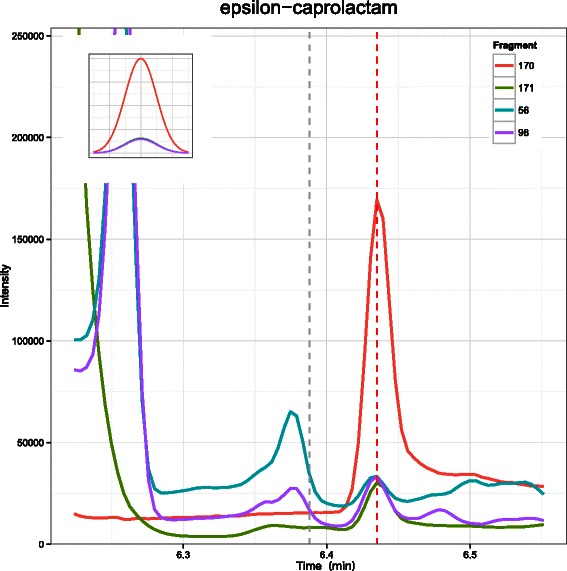
Target EIC. Extracted ion chromatogram (EIC) of monitored fragments for an analyte. Although 56 is considered as the quantifier fragment, 170 is a better choice as suggested by the optimization algorithm in SIMAT

Before advancing to the next steps, we can check the total ion chromatogram (TIC) of the run as shown in Fig. 8:

To find the corresponding chromatographic peaks in the run, we can call getPeak function:

Following that, the extracted ion chromatogram (EIC) of the retrieved chromatographic peaks can be visualized using plotEIC function:

However, the above is done without RI calibration. To adjust for RI, first we call getRI to create a function which can be used to calculate the RI given the RT:

Here, calcRI is a function which accepts RT values as its argument:

Also, calcRI can be used with the getPeak function to redo the data preprocessing, but this time, with RI calibration:

To work with the fragment selection function, i.e. optFrag, we can use the example background library, i.e. Library. This is recommended to be done before the experiment, as after running the experiment, it may not be possible to find an optimum choice among the set of monitored fragments. We can also check the difference between the default set of the fragments, and the ones selected by optFrag function: which gives the mass of fragments as 56, 98, 170, and 171 with relative intensities of 152, 148, 1000, and 156, respectively. On the other hand, we can check the optimization results:

which generates the mass of fragments as 170, 171, 185, and 112 with relative intensities of 1000, 156, 141, and 36, respectively. Although two of the fragments are the same as the original choice, their order has changed after optimization. Also, two new fragments are selected that can be used as qualifiers. As illustrated, Fig. 9 assures us that the fragment with mass of 170 is a better choice as a quantifier for this analyte.

In the example above, the optFrag function is used directly. However, it is usually used within the getTarget function, where the user can set if optimization is desired.

More example data sets are available for testing the tool at http://omics.georgetown.edu/SIMAT.html.

## Conclusions

We introduce a new R package called SIMAT that allows the selection of the optimal set of fragments and retention time windows for target analytes in GC-SIM-MS based analysis. Also, various functions and algorithms are implemented in the tool to: (1) read and import raw data in netCDF format and spectral libraries in NIST MSL format; (2) perform GC-SIM-MS data preprocessing including baseline correction, peak detection and deconvolution, retention index calibration, analyte identification, and quantification; and (3) plot and visualize EICs and TICs, which can be used for manual curation of the retention times or other parameters used for data preprocessing.

Because the tool aims to locate selected ions, the processing time is reasonably short compared to algorithms that look for all possible chromatographic peaks. By using experimental data sets, we evaluated the performance of the method and showed that it is capable of retrieving target analytes with a run time as short as 10min for a data set with 86 GC-SIM-MS runs.

Finally, although SIMAT is tailored to process GC-SIM-MS data, the implemented algorithms can be utilized to search for targets in data acquired in full scan mode. It can also help determine the most appropriate fragment for quantification of analytes in untargeted analyses.

Future work will focus on development of a GUI for the tool. Other improvements in the peak detection algorithms, data visualization, and supporting more input data formats for the raw and library data will be considered.

## Availability and requirements

**Project name:** SIMAT**Project home page:** http://omics.georgetown.edu/SIMAT.htmlhttp://omics.georgetown.edu/http://omics.georgetown.edu/SIMAT.htmlSIMAT.html**Operating system(s):** Platform independent**Programming language:** SIMAT is available as an R package as a free and open-source tool on Bioconductor repository. Moreover, the tool is available as a set of m-files which can be run in MATLAB.**License:** GNU GPL-2**Any restrictions to use by non-academics:** None

## Additional file

Additional file 1
**Parameter settings and compound names including derivatization.** This file includes parameter settings used in MetaboliteDetector and AMDIS. It also includes the name of the compounds in Table 3 with the derivatization as listed in NIST GC-MS library. (PDF 190 kb)

## Abbreviations

AUC: Area under the curve
EIC: Extracted ion chromatogram
TIC: Total ion chromatogram
TMS: Trimethylsilyl
GC-MS: Gas chromatography coupled with mass spectrometry
SIM: Selected ion monitoring, RI: Retention index
RT: Retention time
IS: Internal standard
