# Supplementary material for: SIMAT: GC-SIM-MS data analysis tool
Source: BMC Bioinformatics. 2015 Aug 19;16:259. doi: 10.1186/s12859-015-0681-2 (PMC4539696; doi:10.1186/s12859-015-0681-2)
Supplement: Additional file 1 — Parameter settings and compound names including derivatization. This file includes parameter settings used in MetaboliteDetector and AMDIS. It also includes the name of the compounds in Table 3 with the derivatization as listed in NIST GC-MS library. (PDF 190 kb) [file 12859_2015_681_MOESM1_ESM.pdf]

Supplementary Material for SIMAT: GC-SIM-MS Analysis Tool

1. Parameter settings

In the following sections, we provide the optimized set of parameters used for analysis of SIM data using AMDIS and MetaboliteDetector. These parameters are loosely chosen to maximize the detection rate.

1.1. AMDIS

Type of analysis: Simple/Use retention index data
Component width: 8
Adjacent peak subtraction: Two
Resolution: Medium
Sensitivity: High
Shape requirements: Medium

1.2. MetaboliteDetector

Peak threshold: 3
Minimum peak height: 4
Number of bins per scan: 10
Deconvolution width (scans): 8
Similarity Score: Spec. similarity/Combined score
Max RI difference: 30

2. Compound names with derivatization

In Tables S1, we provide the native compound and derivative names for the compounds listed in Table 2 of the manuscript. These names are obtained from the Fiehn and NIST GC-MS libraries.

Table S1

Index	Fiehn	NIST 2011
1	epsilon-caprolactam	N/A
2	norvaline	N/A
3	valine	L-Valine, N-(trimethylsilyl)-, trimethylsilyl ester
4	urea	Urea, N,N'-bis(trimethylsilyl)-
5	phosphoric acid	Phosphoric acid, tris(trimethylsilyl) ester
6	proline	L-Proline, 1-(trimethylsilyl)-, trimethylsilyl ester
7	threonine	N,O,O'-Tris-(trimethylsilyl)threonine
8	trans-3-hydroxy-L-proline	N/A
9	asparatic acid	Aspartic acid, N-(trimethylsilyl)-, bis(trimethylsilyl) ester
10	phenylalanine	N/A
11	L-glutamic acid-2,3,3,4,4-d5	N/A
12	glutamic acid	Glutamic acid, N-(trimethylsilyl)-, bis(trimethylsilyl) ester, L-
13	lauric acid	Dodecanoic acid, trimethylsilyl ester
14	arabitol	Arabitol, pentakis(trimethylsilyl)-
15	sorbose	L-(-)-Sorbose, pentakis(trimethylsilyl) ether
16	linoleic acid	Linolenic acid, trimethylsilyl ester
17	arachidic acid	Eicosanoic acid, trimethylsilyl ester
